# Launching a Village to promote aging in community: human, social, and financial capital

**DOI:** 10.3389/fpubh.2025.1688002

**Published:** 2025-12-18

**Authors:** Emily A. Greenfield

**Affiliations:** School of Social Work, Rutgers University, New Brunswick, NJ, United States

**Keywords:** organizational development, gerontology, asset-based community development, community capacity and development, civic participation, human capital (HC), social capital, nonprofit

## Abstract

Villages are an innovative, community-based approach to help people thrive as they age. Operating primarily as community-based membership organizations rather than actual “brick and mortar” locations, Villages commonly facilitate services through neighbors-helping-neighbors volunteer programs, offer social events and programs, and serve as a trusted source of information for local resources. Since the first Village launched in 2002, the model has expanded to hundreds of communities, yet the start-up of Villages remains limited in many regions. This paper explores the development period (2022–2025) for one of very few Villages in New Jersey (U.S.). It highlights how local leaders and residents collaboratively mobilized diverse assets—including human, social, and financial capital—to design, develop, and launch the Village. It presents the development work across three phases: establishing the core team and administrative foundations; building the infrastructure; and preparing for launch. Lessons learned include the importance of adopting an asset-based approach and learning through doing, embedding local efforts within larger systems levels, and designing for sustainability and scalability.

## 1 Introduction

In June of 2025, a series of social interactions occurred in a suburban town in northern New Jersey (NJ) that would not have happened otherwise. One community member gave another community member a ride to the dentist. Another community member helped a fellow community member troubleshoot a feature on her mobile device. Two other community members swapped garden tools and stories as one assisted the other with planting geraniums in her yard. And more than three dozen community members—ranging in age from their 40s through their 90s—came together in a neighbor’s backyard to socialize on one of the most pleasant spring nights that year.

These events did not just happen. They came to fruition because of the organized efforts of a group of people working together over the years with a shared commitment to promote vibrant aging in their town. Together, they set out to develop a Village. Their commitment reflects the core ethos of aging in community: people working together to “create mutually supportive neighborhoods to enhance well-being and quality of life for older people at home and as integral members of the community” (1, p. 14).

This paper provides an overview of the start-up of this Village—the Glen Rock Neighborhood Network (https://www.grnnvillage.org/). Villages are an innovative community-driven approach to support residents as they age in place. Most commonly operating as community-based membership organizations and not as physical destinations or singular locations, Villages support aging in community in a variety of ways. They typically facilitate mutual support and neighbors-helping-neighbors volunteer programs, offer community events and programs designed for older adults and intergenerational connections, and serve as a trusted source for information about local resources and services (2). Villages have been celebrated as an innovative place-based approach to promote healthy aging for many reasons, including their promise for helping people maintain a sense of community and purpose as they age, their value proposition for meeting service gaps in cost-effective ways, and their potential to help people with limited family support (3).

It is estimated that there are approximately 300 Villages in the U.S. today, a rapid increase since the founding of the first Village in 2002 (4). Nevertheless, Villages—as a program, organization, and larger social movement—have not yet taken hold in most U.S. states and are emerging slowly in other countries (5). This paper aims to address some of the challenges and opportunities for the development of Villages. It is premised on the idea that case analysis is especially important for advancing social innovations by integrating real-world experiences with theoretical concepts, with strong implications for practice and policy (6).

## 2 Context

Glen Rock is a borough in the western region of Bergen County—a county in the northeast corner of NJ abutting the Hudson River across from New York City. Glen Rock maintains a population of just over 12,000 people, which is smaller relative to most of the immediately adjacent municipalities. According to the 2020 U.S. Census (7), 74.5% of residents identified racially as White alone, 13.3% Asian, 7.01% Hispanic, and 1.3% Black. From 2013 to 2023, the proportion of adults ages 60 and older rose somewhat from 16.2% to an estimated 17.8%, although the proportion of residents ages 18 and younger increased more: from 24.3 to 32.4% (8). Glen Rock also is characterized by relatively high levels of socioeconomic status, with a median household income of $160,417 in 2013 and $210,369 in 2023 (9). Together, these population trends suggest that the community is somewhat gentrifying and becoming younger, which accentuates the importance of initiatives like GRNN to better ensure inclusion and support for older residents who are aging in place.

**Figure 1:**
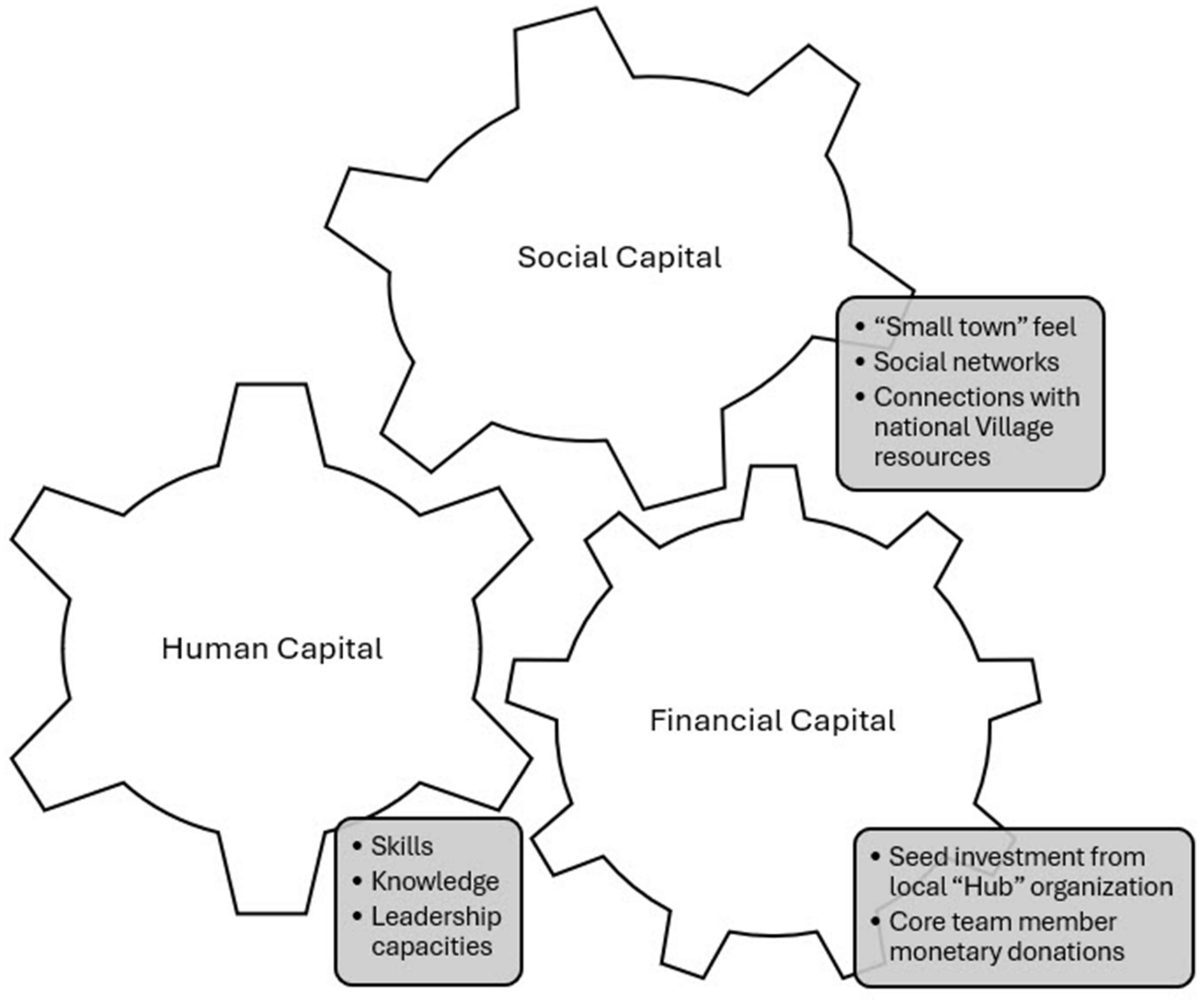
Community capitals leveraged for the initial start-up of the Glen Rock Neighborhood Network (GRNN). This figure displays three types of capital – human, social, and financial – that provided favorable conditions for initially starting the development of GRNN.

Glen Rock has assets of various types that provided favorable conditions for the initial development of the Village. Drawing on concepts from a Community Capitals Framework (10), these assets are described below according to three types: human, social, and financial (refer also to Figure 1).

### 2.1 Human capital

Human capital refers to skills, knowledge, and leadership capacities of individuals, especially those that enhance resources for community development. From the beginning, GRNN had at its disposal several key people to seed the success of pioneering a Village in this region. These individuals worked together over the years as a core group to develop and launch GRNN. Two core group members—who were designated as GRNN co-presidents about mid-way through the development period—brought to the efforts their years of experiences as both long-time residents and locally elected officials. As council members, they had led a community-wide survey about aging in Glen Rock, which provided them with a deep understanding of community needs and priorities. They also brought to the efforts their ability to lead civic groups, troubleshoot interpersonal differences, and co-design systems, policies, and procedures for the public good. The core team also included a community resident [the author], a professor of social work with expertise on aging in community and related models, including age-friendly community initiatives and Villages, as well as a history of cultivating cross-sectoral networks for social change on aging. The group further benefited from the expertise of the Executive Director of North Jersey Villages (NJV), a local nonprofit organization that sponsored and incubated GRNN. She brought to the efforts her knowledge of the national Village Movement, her experience as an advocate for Villages in the region and state, and prior experience in nonprofit development, volunteer management, and aging services. The group also included community residents of various ages and tenure in the community. These residents contributed their skills and life experiences, including their lived expertise of aging in Glen Rock alongside their observations of peers and other community members, as well as their shared commitment to promote aging in place in and through the community.

### 2.2 Social capital

Social capital refers to the value that emerges through connections among individuals and groups. Glen Rock as a community is known to have high levels of social capital, with a “small town” feel including many faith organizations and a broad range of civic groups and voluntary associations. Core team members (refer to “human capital” above) also contributed critical connections and relationships. For example, community residents on the core team engaged their peers and neighbors to engage in early GRNN activities, such as by encouraging them to attend initial GRNN information sessions and subsequent community-building events. NJV helped GRNN connect with resources from the national movement, primarily through Village to Village Network (VtVN), a national nonprofit organization. For example, GRNN benefited from VtVN’s national mentorship program, a Village “101 toolkit” with a sequence of tasks for developing the Village, and a document library and national online forum for Village leaders to address current issues and share timely resources with each other.

### 2.3 Financial capital

Financial capital refers to monetary resources that can be invested in community-capacity building. Although initiating the Village development process did not involve major expenses, such as hiring staff or renting building space, it did involve some tangible resources with associated expenses, such as printouts of materials, refreshments at events, and digital subscriptions (e.g., website hosting and a Zoom account). GRNN benefited from the financial investment of NJV as a regional nonprofit with private donations to spur the development of Villages in the region. Generous donations from several core team members, who understood the importance of additional operating funds to achieve early strategic goals, also supported GRNN’s start-up. These early contributions were important to seed GRNN before the procurement of additional revenues, including membership fees, grants, and other donations later in the development process.

## 3 Program development phases and benchmarks

Work toward GRNN’s development began years before its official launch in April of 2025, marked by the roll-out of its membership program. Beginning in 2016, NJV had identified Glen Rock as one of several municipalities in the region with strong potential to launch a Village. It was not until the winter of 2021–2022 (delayed, in part, because of the COVID-19 pandemic) that NJV helped to convene an initial group of interested residents and community leaders to begin designing the Village. The sections below summarize programmatic efforts toward GRNN’s launch across three phases (refer also to the overview summarized in Table 1). Throughout this section, the author uses the terms “we,” “our,” and “us” to refer to the group of individuals who worked together to develop GRNN, which included the author.

**Table 1:** Overview of GRNN development phases and benchmarks pre-launch.

Phase	Overview	Benchmarks
1	Establishing the core team and administrative foundations (January 2022 – June 2023)	Formation of a work group to begin planning the Glen Rock Neighborhood Network (GRNN) Designate roles and responsibilities between North Jersey Villages (NJV) as the “Hub” organization in support of GRNN as a “Spoke” Village Initiate community-wide outreach about GRNN Establish key operating systems (e.g., collaborative file-sharing, constituent relations management system)
2	Building out the infrastructure (June 2023 – August 2024)	Add information on the GRNN website and build out other digital platforms Expand and activate GRNN committees to develop key programmatic components (e.g., membership policies, volunteer program) Expand community outreach Pursue funding for a part-time coordinator in anticipation of launch
3	Preparing for launch (August 2024 – March 2025)	Complete key tasks and finalize outputs (e.g., membership manual, onboarding forms, volunteer insurance coverage, budget plans) Recruit and onboard a part-time coordinator Offer pre-launch registrant program Recruit, train, and onboard initial pool of GRNN volunteers

### 3.1 Phase 1 – establishing the core team and administrative foundations (January 2022–June 2023)

GRNN first started taking form because of the vision and initiative of two local champions. These individuals, at the time, were elected council members who were leading an age-friendly community survey in Glen Rock for residents 55 years and older. Findings indicated the need for assistance with transportation, a desire for more opportunities for social connection, and residents’ overwhelming desire to remain in Glen Rock and age at home. The then-council-members understhood how an operational Village in Glen Rock could address many of these needs given Villages’ long-standing emphasis on volunteer-based transportation services, social connections programming, and miscellaneous supports for aging in place (2). Complementing their age-friendly efforts in the public sector, they opted to pursue the development of a Village as private residents. They connected with the Executive Director of NJV, and, together, they convened a small group of people, who began meeting regularly to collaboratively design, plan, and eventually launch GRNN.

Our efforts in this early phase focused on three key areas. A primary focus was the establishment of organizational and administrative set-up, with a focus on designating the roles and responsibilities between NJV as the “Hub” organization in support of GRNN as a “Spoke” Village. This work led to GRNN and NJV completing a Memorandum of Understanding (MOU) in June of 2023. The MOU formally designated NJV as the fiduciary and administrative sponsor of GRNN, including the extension of its nonprofit, tax-exempt 501(c)3 status, allocation of start-up budget, insurance coverage, legal assistance, and discounted membership in VtVN. The MOU further specified that GRNN, in turn, was expected to provide reports to and share information with NJV, incorporate NJV’s logo and tagline in marketing and publicity materials, and operate a committee with budget oversight and goal-setting.

A second focus of our work was initiating community-wide outreach about GRNN, primarily through a series of community information sessions. These sessions not only allowed us to connect with interested community members, but it also provided the opportunity to begin developing our branding, communications, and messaging about GRNN. It further served to “test the waters” for people’s interest in supporting or joining a Village in our community. Comments and questions from attendees regarding their potential future engagement with GRNN (e.g., about membership fees and the relationship between GRNN and the municipal authority) helped with subsequent Village design considerations. We also gathered interest forms from all attendees at the sessions, with information through these forms becoming the beginning of GRNN’s contacts database.

Third, we began to establish key operating systems. We procured our main digital platforms, including a website domain and hosting service, a constituent relations management system (CRM), Google Drive for collaborative file-sharing and archiving, Gmail and Google Voice for receiving and sending messages, and a Facebook account. Establishing accounts and, in some cases, paying for services and assigning logins, helped to further establish key roles among core team members and our relationship with NJV.

### 3.2 Phase 2 – building out the infrastructure (June 2023–August 2024)

Phase 2 entailed building our systems and infrastructure from the foundations established in Phase 1. Having gained access to the CRM platform in Phase 1, we next needed to learn how to leverage this software for multiple functionalities. For example, the platform provided a recommended outline for website pages, but populating these pages with information about GRNN required us to settle on key design decisions, such as information about what types of services volunteers would provide, how to donate and donor recognition practices, and criteria for becoming a volunteer. These decisions also were necessary to advance our programming of the back end of the platform, such as setting membership fees and storing information from participant background checks.

Accordingly, in this phase, our core group began meeting more frequently—twice a month rather than once a month. We also activated our emerging committee structure—teams comprising core team members who deliberated on specific operational, procedural, and policy areas in greater depth. For example, one group was activated to develop our membership policies and manual. Another group focused on building out our neighbors-helping-neighbors volunteer program. (Refer to Table 2 for a list and overview of all GRNN committees.)

**Table 2:** Overview of GRNN committees.

Name	Purpose
Core team	This committee meets at least twice a month to provide oversight, planning, and operations for GRNN as a whole. All committees (as listed below) share updates and recommendations to the core team, which then discusses and votes on action items (e.g., policy adoption, resource allocation, etc.).
Outreach team	This committee convenes meetings, organizes events, and advances other outreach efforts to promote GRNN and engage prospective members, volunteers, and community partners. Responsibilities also include managing communications such as flyers, social media, and updating a weekly digital e-blast with community events.
Membership team	This committee focuses on supporting and welcoming new GRNN members through personalized outreach, including responding to inquiries, making friendly follow-up calls, and offering invitations to events. The committee also helps members complete forms, pay fees, and learn how to make the most of GRNN benefits. They also develop recommendations for membership-related policies and benefits.
Volunteer management team	This committee focuses on recruiting, onboarding, and training GRNN volunteers. The committee also makes recommendations for volunteer-related policies and organizes volunteer recognition programs.
Events team	This committee plans and executes GRNN events. They develop ideas for events, as well as organize logistics and conduct outreach and follow-up concerning events.
Data & evaluation team	This committee manages GRNN’s digital tools and subscriptions, ensuring team access, troubleshooting issues, and maintaining data privacy protections. It also includes supporting other committees in using these tools effectively, digitizing information, and generating evaluation reports.
Finance team	This committee works on budget development, monitoring, and bookkeeping. They also assist with purchasing and procurement.
Fundraising & sponsorships team	This committee focuses on developing and managing GRNN’s sponsorship and donor engagement strategies, including policies, as well as systems for acknowledging donors and sponsors. The committee also identifies funding opportunities such as grants and securing support for both general operations and specific initiatives through individual donors and corporate sponsors.

We also continued outreach, adding components beyond the information sessions in Phase 1. We invested in a “tabling kit” (with a banner and handouts), allowing us to have a consistent presence at community fairs and to continue adding people to our database. With support from our then-newly established Events Team, we also began regularly hosting community-wide social events, such as movie matinees and backyard gatherings. These events allowed us to both deepen (i.e., multiple touchpoints with the same people) and broaden (i.e., reach new people) our contacts. These gatherings also enhanced our community-wide presence through our outreach about these events (e.g., social media, flyering, and e-blasts). We also began populating the online calendar of the GRNN website with “events around town” of interest to aging in our community, such as program at local public libraries, civic events, and wellness workshops. We activated a feature in the CRM platform to send a weekly e-blast from GRNN to the entire mailing list with a summary of upcoming events—further enhancing our regular touchpoints with the community.

As we drew closer to launch, we recognized the need for enhanced and consistent administrative support. While our all-volunteer team was able to slowly, yet steadily, work on tasks (e.g., design a brochure, enter information from event sign-up forms into the CRM), we were concerned that not having a point person to respond to inquiries and communicate with GRNN participants in a guaranteed timely fashion would undermine our efforts. We also realized the importance of having some paid staff who could “fill in” on some of the tasks that our core team could not manage at a given point in time (e.g., because of family or paid work responsibilities). Therefore, toward the end of phase 2, we developed a grant proposal to a local private philanthropy for hiring of a part-time coordinator. We also budgeted for subsidizing membership fees for participants with limited incomes as part of this proposal. We positioned the project as not only start-up support, but also as model development: that the successful design, development, and launch of a successful and ultimately sustainable Village in Glen Rock could serve as the foundation for the start-up of other Villages in our region. The success of this proposal, in part, helped us to progress to Phase 3.

### 3.3 Phase 3 (August 2024–March 2025) – preparing for launch

We accelerated our work during the final months leading to GRNN’s launch. This phase involved completing many tasks and finalizing outputs, including the GRNN membership manual, onboarding forms, volunteer training materials, website, and budget plans. We also implemented tasks to bring on a part-time coordinator, including collaboratively developing the position description, recruiting applicants, hiring, and onboarding, including the procurement of a GRNN laptop, mobile device, and cell service. Much of this work was supported through the expansion and solidification of the GRNN committees. We also formally designated chairs for each committee and began to actively recruit additional volunteers to join the teams.

In this phase, we created intentional “on ramps” for people to engage with GRNN in anticipation of launch, both as members and as volunteers. Regarding membership, we developed a pre-launch early interest deposit/donation program. Drawing from our database developed of those expressing interest over the prior years, we invited people to pay a small amount that would entitle them to discounted membership upon launch. The pre-launch registrant program served as a mutual “pledge of support”—a sign of intention among community members to join GRNN and a signal of commitment among GRNN organizers to complete GRNN’s launch. It also served as a basis for budget projections and establishing membership fees.

We further focused on expanding our emerging volunteer program by hosting community-wide volunteer recruitment parties, followed by volunteer training sessions, which covered content such as volunteer procedures, policies, and training scenarios (e.g., “What should I do if a member…?”).

These initial training sessions led to signed volunteer agreements and background checks, resulting in the onboarding of a dozen volunteers “at the ready” upon GRNN’s launch. We also invited volunteers to make donations to help cover the cost of their background checks, reflecting an initial fundraising effort for specific operational expenses.

### 3.4 GRNN metrics post-launch

As of November 1, 2025 (six months following launch), GRNN has 36 active members, three additional members in the process of on-boarding, and 24 trained services volunteers. These volunteers have fulfilled 79 service requests to date for a total of approximately 100 service hours. Of the eligible members who have been part of GRNN for at least a month, approximately 50% have requested at least one service, and 78% of all members to date have engaged in at least one GRNN social gathering (out of six total possible). The most common service request has been transportation assistance (non-medical followed by medical) alongside help in and around the home (e.g., technology assistance, chores around the house). Although GRNN has not yet administered a participant satisfaction survey, member and volunteer feedback on service encounters have been only positive, with no complaints reported to date about any negative experiences from either a member or volunteer (M Silver, meeting 10/30/205 and email 10/31/2025). In addition to membership services volunteers, GRNN has a core team of 15 volunteers who support GRNN operations augmented by the part-time coordinator. Most have joined GRNN as members, and all attend approximately three meetings per month (core team and other committees). Also, GRNN’s mailing list includes 235 unique contacts.

## 4 Discussion

The development of GRNN was a labor of vision, collaboration, patience, and persistence. In addition to the satisfaction and joy we feel for GRNN now as part of the fabric for aging in our community, we have gained many insights, several of which we share below.

### 4.1 Adopt an asset-based approach and learn through doing

Fundamentally, efforts to develop GRNN were in service to creating something that did not yet exist—in our community, in our region, and largely in our state. We consistently came up against resource constraints during the years of our efforts: “If only we had a volunteer who could…” “If only we had part-time staff who could…” “If only there were a tool that could help us…” As we used the “Village 101” start-up checklist, as made available through VtVN and adapted for GRNN by NJV, we often found ourselves at somewhat of a loss when we just did not yet have the capacity to accomplish key tasks.

Nevertheless, we learned through experience not to perseverate on what we could not yet do, but instead, to focus on what we could accomplish, one here-and-now moment at a time. This perspective follows from the general principle of Asset-Based Community Development (ABCD), an approach to community development that emphasizes recognizing and leveraging existing resources and capacities within a community rather than focusing on deficits or unmet needs (11). It also is consistent with a key tenant of Strategic Doing™, which prioritizes working with the resources that a group of collaborators actually have rather than becoming immobilized because of what is missing (12). We recognized early on that it would take time to accumulate the essential resources to garner additional assets (e.g., greater support for information technology and record-keeping), and that patience and embracing “the long view” with our all-volunteer effort was essential.

We also learned over time how to work “smarter, not harder” together. It was through actually doing things together over the years that we better understood each other’s strengths and how to leverage them more strategically to keep moving forward. For example, we learned that high school student volunteers were eager and efficient at posting event flyers in key locations throughout town, which freed up older adult volunteers to help with other duties as well. We learned which core team members were drawn to leading in different ways, such as some who flourished through organizing community events, others who became energized by building out data and information systems, and others who found satisfaction in iterating on policy and procedures documents.

At the same time, we also learned that “together” is not necessarily always “in unison.” From the very beginning, we were advised that there would be disagreements, and that was indeed our experience. For example, core team members, at times, disagreed on the responsibilities of the “Hub” organization relative to the “Spoke” Village, which tech platforms to invest in, and when the timing was right to promote information about GRNN’s efforts. We now appreciate that disagreement, and at times, conflict, is an inevitable part of working on something new, important, and long-term. If team members can get past points of disagreement and then recalibrate through subsequent shared moments of joy and accomplishment, then there is potential for the group to become stronger not in spite of conflict, but because of it.

### 4.2 Anchor in the local yet embed capacity development within larger systems levels

Core to Villages are their hyperlocal focus and grassroots administration: For Villages, community is not merely a place in which they operate, but serve as the engine that catalyzes and sustains them. This principle was very much our experience and part of an asset-based approach (refer to 4.1 above). At the same time, we were acutely aware of the many ways in which GRNN also needed to leverage resources outside of the community. We learned the importance of positioning the development of our Village as part of larger systems efforts.

Most proximally, our Village was seeded and guided through the critical support of NJV, whose mission is to develop “Spoke” Villages throughout the county. Over 5 years prior to our own efforts in Glen Rock, NJV had cultivated infrastructure and resources that were essential for GRNN’s infrastructure. NJV offered an initial organizational set-up, including pro bono legal counsel, 501(c)3 status, and financial infrastructure (e.g., bank account, credit card, initial operating funds, organization insurance coverage). NJV resources allowed us to more readily build and grow GRNN’s infrastructure, such as covering expenses (e.g., refreshments at early information sessions, printing, discounted membership to VtVN, digital subscriptions) and eventually extending volunteer insurance coverage as we prepared for launch. NJV also offered volunteers from its own organizational team with the necessary skills to support our efforts in Glen Rock from the very beginning. NJV further provided guidance on developing GRNN according to key principles, such as not duplicating resources that already exist and developing GRNN policies consistent within the policies of NJV as a not-for-profit and as required by their insurance carriers. To this day, NJV volunteers and Board members are part of the GRNN core team and other committees, fulfilling mission-critical functions such as assistance with volunteer training, grants administration, bookkeeping, information-technology support, and making resource connections for members. And through GrNN’s own development as the first “Spoke” Village in our region, we are, in turn, growing our collective capacity to seed and incubate Villages in other communities. We also continue to explore and revisit the ever-evolving relationship between GRNN and NJV as our mutual efforts develop over time.

At another systems level, we were keen to embed our Village development efforts as part of a growing Age-Friendly Communities (AFC) Movement, especially in our geographic region. Villages are aligned with the broad aspirational goals of AFCs (13)—to make communities better for long and healthy lives. They similarly value inter-organizational collaboration, as well as older adults’ involvement and leadership. Moreover, there is direct overlap in the goals of Villages and AFC initiatives through their shared emphasis on social and civic participation, information and communication, respect and social inclusion, as well as transportation, housing, and community services. We framed our Village development efforts as part of a broader and established network of AFC leaders in our region (refer to https://agefriendlynj.org/ for more information), emphasizing how the start-up of Villages presents another programmatic “on ramp” for AFC progress. Our messaging was that Villages can appeal to some communities more quickly or easily than a participatory planning model alone, and therefore, the successful launch of our Village could strengthen and expand the broader AFC Movement. This premise was the impetus, in part, for GRNN’s grant request to a local foundation, which allowed for the hiring of a part-time coordinator and progress beyond start-up and into our launch.

We remain hopeful that our capacity development as GRNN—in tandem with the capacity developed among NJV and the broader AFC network in our region—will help to inspire public support (municipal, county, state) for incubating and sustaining Villages. To that point, members of the GRNN core team advocated for the inclusion of Villages as part of the NJ Department of Human Services (NJDHS) *Age-Friendly Blueprint,* which offers statewide strategies to make communities more supportive of aging and inclusive of older residents (14). More recently, individuals from NJV and the author advocated for the inclusion of Villages in the forthcoming NJDHS, Division of Aging Services, State Plan on Aging. This action reflects our continued advocacy for state policy leadership and public-private partnerships to seed innovative, community-centered, consumer-driven initiatives such as GRNN.

### 4.3 Design for sustainability and scalability

Our efforts to launch and develop GRNN very much centered on what was believed to be of importance and feasibility for community members in Glen Rock today. At the same time, we remained acutely aware of building something for impact for other communities and for our own community well into the future. Therefore, sustainability and scalability were also important values that guided our work and decision-making.

Sustainability refers to an organization’s or initiative’s long-term ability to fulfill its mission and provide benefits to a community (15). In our case, we were especially concerned with sustaining GRNN as a branded community initiative with its own organizational structure (e.g., core team and committees) and operations (e.g., regular events and programs, trainings, volunteer service provision, continued relationship development with key partners, such as the local library and faith organizations). Our decision to pursue a grant to support a part-time coordinator presented both opportunities and challenges for sustainability. The creation of this position introduced a major budget line for which we must now plan and fundraise. However, we concluded that operating as all-volunteer—especially after launch with active service requests and onboarding—would be highly likely to become unsustainable at some point in time, given difficulties with quickly cultivating and sustaining a deep enough volunteer pool. In short, to optimize GRNN’s launch and “ready” reputation as a well-functioning, high-impact asset for aging and intergenerational connections in Glen Rock, we thought it essential to bring on a part-time coordinator—solidifying our Village as something that would attract additional support through subsequent grants, private donations, and public-private partnerships.

Planning for long-term sustainability has become more of a focus post-launch. This is, in part, because of the receipt of the philanthropic grant, which has required even greater attention to budget modeling, financial tracking, and longer-term fundraising plans. As an example, our team recently approved the procurement of a specialized CRM system for donor development and fundraising. The software allows for tracking revenues and conducting targeted outreach from fundraising campaigns and events, as well as with individual donors and corporate sponsors. We will also soon have completed our first budget year post-launch, providing an opportunity to analyze the cost structure for organizational expenditures (e.g., coordinator salary, event expenses, platform costs) alongside potential cost optimization solutions (e.g., where to allocate expenses against membership fees or private donations). We are also planning to submit a renewal request for continued funding from the private philanthropic funder to further solidify GRNN as a known and valued resource for aging.

Scalability addresses the ability of an initiative to expand its impact following the initial success of a pilot program (16). In our case, we were especially oriented to developing GRNN to facilitate the expansion of Villages throughout other municipalities in NJ. At the same time, we remained both cognizant and concerned of potential “voltage effects.” Voltage effects refer to cases in which an innovative social program is successful in their initial implementation contexts, but then fail to demonstrate sustained effects as the model expands to additional sites (17). As possible, we have tried to design systems, procedures, and resources that can be readily adopted by other communities in their Village start-up efforts. For example, GRNN has taken careful inventory of the digital platforms used for operations, including the Village CRM, a cloud-based video meetings platform, a collaborative file-sharing system, a collaborative password manager system, an e-marketing platform, a listserv service, and more. This information can allow other communities to more proactively plan for and quickly develop this infrastructure for launching their own Village. GRNN also has created digital storage systems that contain records of key materials we have developed throughout GRNN’s start-up, such as outreach flyers, policy manuals, membership forms, partner agreements, and meeting schedules. These “digital binders” are helpful for long-term use within GRNN as key individuals cycle on and off leadership positions, as well as for sharing with other Village efforts in the region. NJV, as an established nonprofit organization dedicated to Villages, as well as GRNN, potentially can further develop these resources into a model replication toolkit and regional replication plan for Village start-up in neighboring communities. The diffusion of such a toolkit is especially promising in our region given the presence of organized networks of age-friendly leaders (refer to 4.2 above).

## 5 Acknowledgement of any conceptual or methodological constraints

A primary limitation of this “Community Case Study” is that it is based on the perspectives of its sole author, whose involvement on the GRNN development team has been primarily in her capacity as a community volunteer—not as an academic research partner. While other GRNN core team members provided feedback and confirmed the veracity of this paper’s contents, their perspectives were not systematically collected nor analyzed through formal empirical methods (e.g., interviews, surveys, focus groups), nor as part of community-based participatory research. As a result, this article falls within the category of non-research case study (18).

Nevertheless, its insights can help to inform future research studies—including comparative case methods involving Villages across multiple sites—to advance deeper and more generalizable knowledge on the start-up of Villages. For example, the Community Capitals Framework can be used for selecting theoretically diverse cases, reflecting Villages in development with varying degrees of initial types of assets (human, social, financial). Insights from this case study also highlight the importance of prospective studies to be developed over multiple years spanning successive phases of organizational development. Continued advancements in this nascent area have strong potential to amplify “bottom up” innovations like Villages for broader social change on healthy aging—in, with, and through our communities.

## Data Availability

The original contributions presented in the study are included in the article/supplementary material. Further inquiries can be directed to the corresponding author.
